# ‘They don’t want them to have capacity’: Multi‐agency operationalisation of the Mental Capacity Act 2005 in England with adults who self‐neglect

**DOI:** 10.1111/hsc.13839

**Published:** 2022-05-23

**Authors:** Elaine Aspinwall‐Roberts, Valerie Fleming, Rose Khatri, Paul A. Jones

**Affiliations:** ^1^ Faculty of Health, School of Nursing and Allied Health Liverpool John Moores University Liverpool UK

**Keywords:** assessment, mental capacity, multi‐agency working, safeguarding adults, self‐neglect

## Abstract

The number of adults who self‐neglect and thus fall under the aegis of local authority adult safeguarding procedures in England has increased substantially since the introduction of the Care Act 2014. The requirement for collaborative working between agencies dealing with these adults in a safeguarding context is explicit in government policy and legislation. Decisions made by the multiplicity of agencies that may work with people who self‐neglect are largely guided by the Mental Capacity Act 2005 (MCA). The overall objective of this research was to develop a clearer understanding of how the range of agencies that might typically be involved in the life of a self‐neglecting person work together. This article examines how agencies put the MCA into practice in their work with people who self‐neglect, and how they understand their own and others’ roles and responsibilities in so doing. This qualitative study took place in two local authorities in England from 2016 to 2017 and informed a wider action research study which was completed in 2019. Non‐probability purposive sampling was used to recruit participants from the professional groups who might typically be involved with self‐neglect cases. À total of 245 participants from across 17 different professional groups took part in semi‐structured interviews, in a group, paired or individual format, decided by their customary working configuration. Data from the interview transcripts was analysed using thematic analysis. Three key themes in relation to how participants understood the MCA and multi‐agency working emerged from the analysis of this data set. These were; a lack of understanding of the MCA by participants and other agencies; a reluctance to engage with MCA assessments; and a perception of manipulation of the MCA by other professionals. This study underlines the importance of the informed application of the MCA in working with people who self‐neglect, and an urgent need to consider how this could be enhanced if the service user is not to experience intrusive interventions resulting from professional misinterpretation.

**What is known about this topic**

- Numbers of adults who self‐neglect are increasing in England, and will continue to increase due to demographic changes.
- A wide range of professionals may be involved in the care of a person who self‐neglects.
- The status of the self‐neglecting person in relation to the mental capacity to make their own decisions, assessed under the MCA, is key in determining outcomes for the person.

**What this paper adds**

- An understanding of the views of professionals from a wide range of agencies involved in supporting people who self‐neglect, in relation to mental capacity.
- An identification of specific difficulties with the application of the MCA within and between agencies.
- A recognition that these difficulties may impact negatively on both multi‐agency working and the person who self‐neglects.

## 1 INTRODUCTION

In 2005, the Mental Capacity Act (MCA) was introduced in England and Wales, providing a framework to use when assessing whether adults have the mental capacity to make their own decisions, and deciding how to proceed when they are unable to do so (Social Care Institute for Excellence, 2020). Subsequently, the Care Act 2014 (CA) imposed a duty on local authorities to make statutory safeguarding enquiries where an adult with care and support needs is believed to be ‘experiencing, or at risk of, abuse or neglect’ (s42.1), including in cases of self‐neglect. Safeguarding statistics (NHS Digital, 2021) show that local authorities in England completed nearly 13,000 S42 enquiries in the year 2020/21, where self‐neglect was the primary issue, a 26% increase from the previous year.

The CA also requires local authorities and relevant agencies to work in partnership and ‘co‐operate generally’ (CA, s6). The two Acts are designed for different purposes but work together on the ‘front line’ of practice (Pritchard‐Jones, 2020) where health and social care organisations are legally compelled to co‐operate on cases of self‐neglect which require a safeguarding response. An assessment of mental capacity is likely to be key in deciding on whether and how to intervene in such cases.

### 1.1 Application of the Mental Capacity Act in multi‐agency practice

Research is somewhat sparse about how the MCA is applied in practice (Hinsliff‐Smith et al., 2017; Jayes et al., 2021), and how assessments are undertaken (Rogers & Bright, 2019). Much extant research has focused on health staff in clinical settings (Hinsliff‐Smith et al., 2017; Marshall & Sprung, 2016), however, the MCA allows for a wide variety of professionals to assess capacity, according to the decision being made. Brammer and Pritchard‐Jones (2019) argue that many practitioners in the multi‐agency arena continue to be unclear about who is responsible for completing capacity assessments. Hinsliff‐Smith et al.’s (2017) systematic review of the application of the MCA in healthcare practice identifies poor knowledge and understanding of the MCA and tensions in applying it in everyday practice. Scott et al.’s (2020) systematic review of the literature addressing practitioners’ knowledge and experiences of the MCA identifies struggles associated with the subjective nature of mental capacity work and the difficulty of producing objective assessments. Such difficulties have existed with the MCA since its inception (MHF, 2010). The MHF (2010) survey identified that many professionals chose to assess capacity because they thought the service user was making a bad choice, and Jayes et al. (2019) confirmed a continued propensity for professionals to make capacity judgements based on service user characteristics.

In 2014, a House of Lords Select Committee (HoLSC, 2014) subjecting the MCA to post‐legislative scrutiny, described it as ‘a visionary piece of legislation’ (HoLSC, 2014, p. 6), hampered by poor awareness and understanding, whose rights and duties ‘were not widely followed’ (ibid). There is every indication to suggest that these concerns about misunderstanding and misapplication continue (Jenkins et al., 2020). Reports from Safeguarding Adult Reviews have consistently criticised capacity decisions and multi‐agency working (Preston‐Shoot, 2020; Preston‐Shoot et al., 2020). Meanwhile, the MCA Code of Practice (2007) is criticised as being insufficiently clear (Kane et al., 2021) and failing to align with actual practice (Rogers & Bright, 2019).

### 1.2 Self‐neglect and mental capacity

The MCA is key to determining multi‐agency understandings of, and responses to, people who self‐neglect, providing, as it does, a framework for practitioners to establish decision‐making capacity in adults, including those who self‐neglect. Prior to its implementation, Lauder et al. (2005) noted that the lack of a test to establish capacity was a hindrance to practitioners. The MCA framework should now be fundamental to all adult health and social care practice (Ruck Keene, 2021), leading to clearer multi‐agency decision making, as the perceived capacity status of the self‐neglecting person essentially dictates the professional response (Rogers & Bright, 2019). However, the MCA seems, in practice, to have led to confusion about when to intervene (Braye et al., 2014; Hinsliff‐Smith et al., 2017; Shepherd et al., 2018). It is argued that the MCA is widely misunderstood (Jenkins et al., 2020), misapplied and ‘used against people’ (Burgess, 2017, np), and can result in questionable decisions (Ruck Keene et al., 2019).

Self‐neglect presents practitioners with extremely challenging situations in relation to mental capacity, particularly where an apparently capacitous person refuses services (Martineau et al., 2019; Preston‐Shoot et al., 2020). This is potentially compounded by the requirement for multi‐agency working, where it is argued (Cameron, 2016) that scant attention has been paid to significant differences between professions, imperilling the success of working together. A ‘wicked mess’ may then ensue (Hancock, 2010, p. xiii) when self‐neglect is the issue, multi‐agency working is a requirement, and the MCA is the driving legal imperative.

This research explores the nature of the tensions at the nexus of multi‐agency operationalisation of the MCA in safeguarding practice with people who self‐neglect. It expands existing knowledge about how different professionals view the way the MCA is operationalised by other agencies with whom they must co‐operate, in relation to safeguarding people who self‐neglect.

## 2 METHODS

**FIGURE 1:**
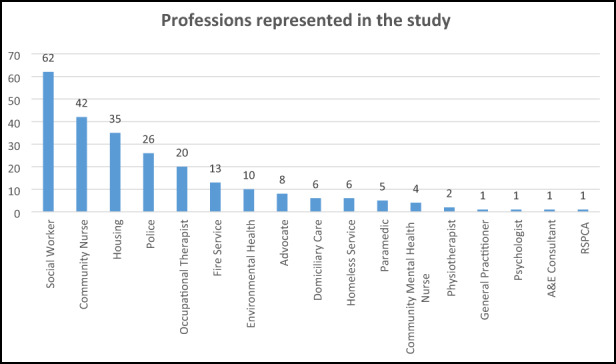
Professions represented in the study

The findings reported here are from the problem‐sensing phase of a wider professionalising action research (Hart & Bond, 1995) study that took place with two local authorities in England between 2016 and 2019. Via the Local Safeguarding Adults Board (LSAB) in each authority, gatekeepers were identified from LSAB member organisations, representing many of the professional groups who might typically be involved with self‐neglect cases (Mason & Evans, 2020), as suggested by practitioners in a pilot study for this research. Gatekeepers were asked to cascade information about the study to their front‐line staff teams, who could then decide whether to participate. Where the organisation was not a member of the LSAB, organisations were approached directly. Non‐probability purposive sampling was used (Doody et al., 2013), which aims to generate insight and in‐depth understanding of a topic (Braun & Clarke, 2013). A total of 245 participants were recruited, with 17 different professions represented (see Figure 1). This study received full ethical approval from the University Research Ethics Committee and from participating organisations where this was required by them.

### 2.1 Data collection

A total of 29 uni‐professional ‘natural group’ interviews (Green & Thorogood, 2014) were held. Differing from focus groups, natural groups were not brought together to meet sampling criteria (Madill & Gough, 2008), but utilised established teams. This allowed access to the interaction between participants and gave insight into how knowledge in their specific setting was produced (Green & Thorogood, 2014) and how meanings were constructed in situ (Kamberelis & Dimitriadis, 2013). A total of 4 paired and 13 individual interviews were also completed, where the participant worked in a very small team or alone. Guest et al. (2017) argue that using two configurations is acceptable if the data collector, the instrument, and the interview environment are consistent. The first two were completely consistent, with each of the individual and group interviews completed by the lead researcher, EA‐R, and all using the same semi‐structured interview format. There was, however, considerable heterogeneity in the interview environments, as these took place in the participants’ workplaces. All participants in the study, irrespective of profession, had an experience of or were currently working with, people who self‐neglected. All participants gave written consent.

### 2.2 Data analysis

Interviews were audio‐recorded and transcribed verbatim. A reflexive thematic analysis led by EA‐R was used to analyse the interview transcripts, following Braun and Clarke’s (2013) guidelines which are flexible, accessible, and useful both for a participatory research paradigm and for a large data set. Transcripts were read repeatedly, and initial coding was undertaken by EA‐R. Preliminary themes were developed, discussed and agreed upon by the research team to ensure confirmability and minimise researcher bias, and refined into a thematic map. Individual themes were then explored further by the team and this paper reports on a specific theme relating to the MCA and the sub‐themes which were developed from that and incorporated into the overall thematic map (ibid). Data were analysed using NVivo 10.

## 3 FINDINGS

Three key themes emerged from the analysis of this data set: lack of understanding of the MCA; reluctance to engage with MCA assessments; and perceptions of manipulation of the MCA. Participants are identified by their professional title, and whether they participated in a group interview (GI), paired interview (PI), or individual interview (II). The number indicates how many interviews took place in this format with this professional group. For example ‘Social Worker, GI 3’ indicates that the speaker is a social worker from the third group interview held with this professional group.

### 3.1 Lack of understanding of the MCA

It was clear that mental capacity and using the MCA, in relation to people who self‐neglect, was a huge challenge for practitioners who took part in this study:

Interviewer: What’s the significance of whether someone who self‐neglects has capacity?

Respondent: Well, it’s the sun and the moon really. (Occupational Therapist, GI1)

Many groups openly discussed their own lack of understanding of the MCA. In the words of one participant, ‘it makes me dizzy speaking about it’ (Community Nurse, GI5). Despite the fact that the MCA came into force 14 years ago, it was described as ‘a massive problem for workers’ (Community Mental Health Nurse, II1), ‘hard to understand’ (Domiciliary Care, PI1), and ‘blurred’ (Psychologist, II1). Some participants found the assessment of capacity a rather mysterious process and were not sure how it was done:

Respondent 1: I thought they had to go to the hospital to get it.

Respondent 2: No, I think somebody just comes out and has a chat with them [service user].

Respondent 3: Yes, my client has never been to the hospital, so I know for a fact they don’t go into hospital. (Housing Officer, GI4)

Others felt that it meant that real problems were missed, and common sense overruled because practitioners focussed solely on capacity:

I think it’s just that legal side of it that’s just become, I feel, a bit top‐heavy, and we’re missing the point of ‘actually, we have got a patient here that’s not eating and drinking, or not washing, going out’, and it gets missed because we’re banging on about capacity. (Community Mental Health Nurse, II1)

Several groups inadvertently expressed misunderstanding of the MCA in the course of their discussions, with some incorrectly eliding the MCA and the Mental Health Act 1983. Some participants were uncertain whether universal or decision specific capacity was assessed (the MCA is clear that it is the latter), and in several interviews the interviewer was asked questions about how the MCA worked.

It was generally agreed by participants that the MCA was difficult for all agencies to operationalise:

it’s across the board, mental capacity’s a problem everywhere, nobody seems to be able to decide on it. (Fire Services, GI2)

However, individual agencies usually believed that *they* were using the MCA correctly. The problem was that others in the multi‐agency picture were not, for example:

Health seem to be culprits not following the principles of the MCA in terms of just assuming they haven’t got capacity … we’re quite stringent, in the way we apply the test. (Social Worker, GI3)

### 3.2 Reluctance to engage with MCA assessments

An elaborate and confusing picture emerged of which agencies did and did not carry out MCA assessments. Decisions appeared to have been arrived at informally, by tacit agreement, often within the particular team or service who did not feel it was appropriate for them to do the assessment:

Then the district nurse was like ‘well, you do the capacity assessment’, but really it’s about accommodation, so should we? So it was very much a dispute in terms of should the nurse and I do it, or should the GP do it? Should someone else do it? (Occupational Therapist, GI2)

The police officers and fire services personnel who took part in this study were clear that they did not carry out capacity assessments. However, paramedics did carry out MCA assessments, which could lead to them feeling very exposed:

I personally feel we are quite vulnerable assessing capacity as paramedics we aren’t social workers, we aren’t doctors, to assess someone’s capacity I think is quite a big thing to do. (Paramedic, GI1)

In the community, social workers felt that they tended to carry out the majority of assessments and one social worker (GI5) argued that this was because ‘no other agency particularly *wants* to do capacity assessments’. It became evident from other groups that this was indeed the case. Reasons given by other professionals for reluctance to undertake assessments included insufficient remuneration, training, expertise or qualifications, or simply being unwilling to take on the responsibility. However, even where staff had reasons for not doing assessments, some remained irritated and frustrated that they were ‘not even in the room’ (Housing, GI2) when they felt they had important information to contribute. Several groups identified a feeling of ‘snobbery’ or ‘preciousness’ from other agencies towards their profession, which inhibited their participation in assessments. One Housing Officer (GI3) felt they were seen as ‘just the thickos’. Conversely, other participants were frustrated by being asked to carry out assessments when they did not know the person at all:

We’re going in as a one‐off to do a capacity assessment, it’s wrong it should be someone who knows that person better. (Social Worker, GI5)

For some groups, their lack of expertise meant that they found it difficult to challenge the capacity decision of others, even if they had chosen to forgo gaining the expertise to be able to do this:

But we aren’t the experts in it, so, I think we feel like we are fighting a losing battle with it, because we aren’t the experts. (Housing Officer, GI4)

Participants were confounded by the capacity conclusions that other practitioners came to, particularly where the term ‘lifestyle choice’ was used to explain a deleterious situation. Participants discussed their feelings about capacity assessments done by others, often expressing disbelief that capacity was found:

And you’re thinking ‘how on earth has this woman got capacity?’. (Fire Services, GI2)

It was felt that some agencies only had minimal knowledge and, therefore, could not make robust decisions:

They [other professionals] will say ‘oh, they’ve got capacity, and I know they get an hour’s training. (Homeless Service, PI1)

However, some groups who declined to undertake capacity assessments themselves, were nonetheless happy to challenge those who did:

We’re not trained at all in terms of capacity but in terms of challenging, alright the person’s got capacity, however, we’re still telling you we’ve identified a problem, capacity or not, there’s still an issue there. (Fire Services, GI1).

### 3.3 Perceptions of manipulation of the MCA

A recurrent belief expressed by interviewees was that other agencies deliberately manipulated MCA assessments to suit their own agency agendas and financial situation. This manipulation could work in two ways, according to whether it was perceived that other agencies wanted or did not want self‐neglecting people to have capacity.

#### 3.3.1 Agencies wanting people who self‐neglect to be found to *have* mental capacity

Various reasons were articulated to support the belief that some agencies actively wanted service users to have the mental capacity to make decisions, because it permitted inaction by the assessing agency (often social services):

It was a gift to some people, the legislation saying that capacity will be assumed unless you can prove otherwise. (Housing Officer, GI3).

The belief that the MCA was used to further the objective of saving money was expressed by several groups, with the suggestion by some that ignoring signs of self‐neglect was a deliberate ploy. One group of community nurses related it to a wider political agenda and unstated financial constraints:

R1: It’s very political really though isn’t it, capacity, because incapacity costs. And if it’s going to lead to care, and if somebody’s ticking along in their own home, and …

R2: I think sometimes it’s a bit of a cop out as well, oh, he’s got capacity …

R1: If somebody is borderline, isn’t it easier to say ‘no, they have got capacity’ let’s just tick along with community nursing on this one because we are NHS’ (Community Nurses, GI2).

The capacity ‘cop out’ was felt to be used to ‘dismiss people’ (Homeless services, PI1), and to allow other agencies to withdraw:

Because it closes down an avenue of action that might be able to be taken to help that situation, and all eyes look to you, ‘well, they’ve got capacity, so it’s over to you, deal with it. (Environmental Health Practitioner, GI1).

This led to a feeling that it was pointless to refer people who self‐neglected to social services and other agencies because having mental capacity gave a ‘green light’ (Fire services, GI1) to them to decline to act, which could result in a farcical cycle:

It always comes back, ‘no, there’s nothing we can do, they’ve got capacity, just got to let them get on with it’ and then it just keeps revolving and revolving, we keep putting the referral back again and it comes back, bing bong! (Community Nurses, GI1).

#### 3.3.2 Agencies wanting people who self‐neglect to be found to *lack* mental capacity

Conversely, other participants (mainly, though not only, social workers) held the view that other agencies wanted people who self‐neglected to *lack* capacity:

I’d say every single case I’ve had, that has had someone that self‐neglects, ‘they haven’t got capacity’, in regards to some professionals’ opinions. It’s every case, not just occasionally (Social Worker, GI5).

Reasons given for wanting people to *lack* capacity were again predominantly financial:

It’s almost a kind of, they feel it’s a tick box, ‘well, they were neglecting themselves at home, they haven’t got capacity, so surely you have got to look at placement (Advocacy Service GI1).

However, a finding of lack of capacity was also a way of abdicating responsibility:

It’s passing the responsibility, that’s what it is. As soon as you say they haven’t got capacity, somebody’s got to take responsibility for them… (Fire Services, GI2).

Lack of capacity could also permit intrusive intervention. One group of social workers (GI3) argued that where capacity was lacking it was construed by others as a ‘done deal’, whereby if lack of capacity could be found, then anything could be done to the service user:

‘Especially around self‐neglect though, because they [other professionals] just think ‘well, they’re not washing themselves in the community, so just chuck them in 24 hour care’. (Social worker, GI3).

It could also be the easy way out, though still leading to conflict:

… you know, if we say that person hasn’t got capacity, it’s almost easier when they haven’t got capacity, when you do the assessment, and you go, ‘result ‐ no, they haven’t got capacity, so let us all charge in here and let us all squabble’. That’s an easy scenario. (Community Nurse, GI2).

## 4 DISCUSSION

The House of Lords post‐legislative scrutiny of the MCA argued that the culture of paternalism in health, and risk aversion in social care, had prevented the Act ‘from becoming widely known or embedded’ (HoLSC, 2014, p. 6). However, this study found that the Act is very well known in multi‐agency working with people who self‐neglect, and it causes apprehension and conflict. Its embedding appears to have become distorted, resulting in a practice that, rather than being person‐centred, has become professional agenda led.

### 4.1 Lack of understanding of the MCA

The finding of a lack of understanding of the MCA has been identified by different studies involving diverse service user groups (e.g. Shepherd, et al., 2018), and by public bodies (e.g. CQC, 2019, 2020). However, the MCA came into force in October 2007, more than 14 years ago. This indicates that attempts to widen understanding of its use have not been altogether successful. The primary recommendation of the HoLSC (2014) report was the formation of a body to oversee the implementation of the MCA. The National Mental Capacity Forum was subsequently established, but this research found no awareness of this body amongst participants.

Providing further training would appear to be one obvious solution. It was clear in this study that lack of training was a key issue, and this was remarkable given the length of time the Act has been in force (Alonzi et al., 2009). Some participants reported having no training at all, and there was a high level of misinformation about the Act. However, both Rogers and Bright (2019) and Willner et al. (2013) found that the benefits of training in the MCA 2005 might be limited, and may not lead to changing practice (Jenkins et al., 2020). Training appears to raise awareness about the MCA 2005 but does not seem to make practitioners any more able to apply it practically. Although £8.65 m was made available to local authorities between 2006 and 2008 to provide MCA training to their own staff and partner organisations (Taylor, 2015), it appears it may have been only a partial success and is unlikely to have been maintained at earlier levels, with newer staff, and changed priorities, such as the reform of DoLS (Taylor, 2015). This study showed that there is a continued need, and thirst, for more discussion and learning on the MCA. However, this is not necessarily a need for training per se, but for the opportunity to discuss fears and confusion, to admit to uncertainty in a non‐threatening forum, and to try to build a common sense of purpose (Cameron et al., 2014). The use of virtual forums, which have become commonplace during the pandemic, could be usefully explored here (Manthorpe et al., 2021).

### 4.2 Reluctance to engage with MCA assessments

In much of the MCA implementation literature there is an assumption that professionals will be confident, indeed enthusiastic, about carrying out mental capacity assessments. It is, after all, a chance to ‘have a real conversation with the person on their own terms and applying their own value system’ (Ruck Keene et al., 2019, p. 3). What emerged from this study was genuine confusion about which agencies should carry out MCA assessments (Hinsliff‐Smith et al., 2017; Ratcliff & Chapman, 2016) coupled with considerable reluctance by some agencies to take on responsibility for completing them. An elaborate picture emerged of participants who opined that they did not, should not, could not, or would not carry out assessments. Participants from several agencies stated categorically that they did not carry out MCA assessments, a position without justification under the current legal framework in England. The Act itself does not specifically include or exclude any professional from carrying out the assessment. The MCA was designed to move away from a need for expert judgement, as required, for example, by the Mental Health Act 1983, to mean that a wide range of people could be involved in the assessment of mental capacity. The current advice from the Social Care Institute for Excellence (2017, np) states that **‘**Anyone caring for or supporting a person who may lack capacity could be involved in assessing capacity’. The SCIE is clear that ‘good professional training is key’ (ibid) rather than reliance on experts.

Generally, it is the person who is proposing to take the step in question (for which a choice needs to be made) who should carry out the assessment. This will depend on the decision to be made and, ideally, the practitioner with the best knowledge of the person being assessed. As Ruck Keene et al. (2016) argue, the capacity assessment will be more robust where it is done by those who know the person best. This study found that this was simply not happening for service users who self‐neglected, who were passed round agencies like ‘hot potatoes’ (Social Worker, GI2).

In this study, paramedics and social workers reported being frequently ‘parachuted in’ (Ruck Keene et al., 2016, p. 4) to do ‘snapshot’ (ibid) assessments. Social workers, whilst sharing this frustration, were generally clear about the requirements of the MCA and not under critical time pressures. Paramedics, however, felt very vulnerable, particularly as they perceived that a wrong decision could lead to an assault charge or the loss of their professional registration. Jones et al. (2014, p. 180) note that ‘Police officers and paramedics often share constant challenges in real‐time situations relating to consent and capacity’. However, the sharing of challenges was not evident here, as the police officers who participated in this study were clear that when capacity was an issue they would usually ask paramedics to carry out the assessment. The third ‘blue light’ service, Fire Services, also did not see it as part of their remit to carry out capacity assessments. The issue of the responsibilities of the emergency services for assessing mental capacity, or at the very least participating in such assessments, is one that needs much further exploration.

This study showed that although many professional groups were unwilling to shoulder the mental capacity responsibility, they were nonetheless highly critical of those who did, leading to considerable conflict between agencies. Although there is as yet little research in this area, there are indications that problems with the mental capacity assessments of others can be very significant. For example, Ruck Keene et al. (2019, p. 64) in analysing capacity cases dealt with by the Court of Protection, found that 40% of cases involved a dispute between professionals.

This presented a contradiction in terms of reluctance to engage with the MCA as described above. Although participants felt frustrated by the capacity decisions of others, they did not choose to enhance their professional status by seeking to be equipped to carry out capacity assessments. To consider doing this was described as ‘overstepping’ (Housing Officer, GI1). They were, however, content to criticise how assessments were done and the decisions that were reached by others (Clerk et al., 2018). No participants were of the view that they would offer information spontaneously, but then they were irritated when they were not asked. Participants rarely considered doing assessments in conjunction with other professionals. These could be seen as self‐defeating stances, which ultimately impact negatively on the service user and which are a distortion of the original purpose of the Act (HoLSC, 2014).

### 4.3 Perceptions of manipulation of the MCA

Many participants in this study voiced the belief that other professionals manipulated capacity assessments. It was not whether the service user themselves actually had or lacked capacity that was the crucial determinant, but whether the agency involved ‘wanted’ or ‘did not want’ them to have capacity. This study found this perception to be embedded into practice, where a distorted application appeared to have become an entrenched heuristic device. Rather than being the perceived capacity status of the person who is self‐neglecting that dictates how professionals will respond (Rogers & Bright, 2019), this study suggests that in multi‐agency practice it is the pressures upon professionals that will dictate how they respond. Participants were effectively suggesting that the assessment of mental capacity was being manipulated for organisational reasons, to suit their agency agendas and financial situation rather than being based on the abilities of the individual involved. It was being indirectly argued that the MCA has been pressed into the service of the austerity agenda in relation to self‐neglect. This would seem to strike at the professionalism of the practitioners involved and the veracity of their assessments. Rather than being risk averse (HoLSC, 2014), social work staff were very often perceived as disregarding risk in their urge to ‘find’ capacity and therefore avoid financial outlay.

Other groups, predominantly social workers, held the view that other agencies *wanted* people who self‐neglected to *lack* capacity. Some of the reasons given were the same as the reasons for wanting people to *have* capacity and were predominantly financial. The belief that the MCA was used to further the objective of saving money was expressed by several groups, with the suggestion that this was a quite deliberate ploy.

A finding of lack of capacity suggested a way of abdicating responsibility to another agency, rather than permitting inaction by *any* agency, as where capacity was found. It also permitted intrusive intervention. The two worked together when it came to decisions about moving someone into a care setting, whereby if they did not have the capacity, it was expected social services would remove the person from their home, and other agencies were excused further responsibility.

## 5 STRENGTHS AND LIMITATIONS OF THE STUDY

This study is strengthened by the occupational span of its participants, who sustained their involvement throughout the research. As with any interviews, there was a risk of participant bias, in that those who volunteered to participate could have been those with strong positive or negative views on the issues covered.

There was good occupational distribution, ensuring strong multi‐agency representation. However, some professionals who could play an important supporting role with people who self‐neglect, such as community pharmacists, could not be recruited within the timeframe, and others, such as GPs were under‐represented.

This was locally based research, and even within the two local authority areas from which participants came, there were many differences, and some idiosyncrasies, in local policies, procedures and resources. These limitations may restrict the ability to transfer these findings to other geographical areas, where service availability or the priorities of the elected members of the authority may differ. Finally, the interviews reported here were completed before the Covid 19 pandemic. It is possible that the pandemic will have increased agencies’ willingness to work together more profitably (Manthorpe et al., 2021).

## 6 CONCLUSION

This study suggests that some professional groups perceive that there is potential for assessors from other professions to tailor the capacity assessment outcome depending on the priorities of their employing agency. This amplifies the anecdotal findings heard by the Select Committee (HoLSC, 2014, p. 51) where it was argued that the presumption of capacity could be used to justify lack of intervention, either erroneously or deliberately. The findings of this study move beyond that to suggest a belief by professionals in the deliberate manipulation of capacity assessments by other agencies to justify actions and facilitate resource rationing, with agencies felt to be assessing capacity guided by their own agenda rather than by the presentation of the self‐neglecting person.

This study also finds that there remain significant problems with the application of MCA, which may have the potential to be magnified and distorted within the multi‐agency arena. Kane et al. (2021, p. 3) identify a ‘translation gap’ between the actual criteria set out for the assessment of capacity in the MCA, and the reality of practice. This study suggests that not only does such a gap exist, but that it is potentially widened by ignorance, reluctance, mistrust and perception of manipulation of the capacity legislation by professionals. On a wider level, professional struggles with the exigencies of the MCA could be seen as exemplifying the contradiction inherent in balancing the safeguarding of vulnerable self‐neglecting people with attempting to promote their autonomy.

Most importantly, the self‐neglecting service user is the one who ultimately is at risk of experiencing harm, either through abandonment by professionals in the guise of promotion of autonomy (Flynn et al., 2003) or highly intrusive intervention in the name of benevolent protection. This harm may be inadvertently caused by practitioners who all believe that they are simply doing their job.

## AUTHOR CONTRIBUTION

All listed authors have contributed to the manuscript and have agreed to the final submitted version.

## FUNDING INFORMATION

LJMU provided the funding for Elaine Aspinwall‐Roberts to undertake the PhD study from which the findings of this article are taken.

## CONFLICT OF INTEREST

The authors are not aware of any conflicts of interest.

## ETHICAL STATEMENT

This study received full ethical approval from Liverpool John Moores University Research Ethics Committee (Ref no: 15/EHC/105), and from participating organisations where this was required by them.

## Data Availability

Data are available on request from the authors.

## References

[hsc13839-bib-0001] Alonzi, A. , Sheard, J. , & Bateman, M. (2009). Assessing staff needs for guidance on the mental capacity act 2005. Nursing Times, 105(3), 24–27. 19248375

[hsc13839-bib-0002] Brammer, A. , & Pritchard‐Jones, L. (2019). Safeguarding adults (2nd ed.). Red Globe.

[hsc13839-bib-0003] Braun, V. , & Clarke, V. (2013). Successful qualitative research. Sage.

[hsc13839-bib-0004] Braye, S. , Orr, D. , & Preston‐Shoot, M. (2014). Self‐neglect policy and practice: Building an evidence base for adult social care . https://www.scie.org.uk/self‐neglect/policy‐practice/evidence‐base?gclid=EAIaIQobChMIoLvRnIec8gIVbIBQBh1wSgFjEAAYASAAEgJj5fD_BwE

[hsc13839-bib-0005] Burgess, I. (2017). The mental capacity act 10 years on: ‘It is still misapplied and used against people’. *Community Care*. https://www.communitycare.co.uk/2017/09/27/mental‐capacity‐act‐10‐years‐still‐misapplied‐used‐people/

[hsc13839-bib-0006] Cameron, A. (2016). What have we learnt about joint working between health and social care? Public Money & Management, 36(1), 7–14. 10.1080/09540962.2016.1103411

[hsc13839-bib-0046] Cameron, A. , Lart, R. , Bostock, L. , & Coomber, C. (2014). Factors that promote and hinder joint and integrated working between health and social care services: A review of research literature. Health and Social Care in the Community, 22(3), 225–233. 10.1111/hsc.12057 23750908

[hsc13839-bib-0007] Care Quality Commission . (2019). The state of health care and adult social care in England 2018/19 . https://www.cqc.org.uk/sites/default/files/20191015b_stateofcare1819_fullreport.pdf

[hsc13839-bib-0008] Care Quality Commission . (2020). The state of health care and adult social care in England 2019/20 . https://www.cqc.org.uk/sites/default/files/20201016_stateofcare1920_fullreport.pdf

[hsc13839-bib-0009] Clerk, G. , Schaub, J. , Hancock, D. , & Martin, C. (2018). A Delphi survey of practitioner’s understanding of mental capacity. The Journal of Adult Protection, 20(5/6), 174–186. 10.1108/jap-05-2018-0009

[hsc13839-bib-0010] Doody, O. , Slevin, E. , & Taggart, L. (2013). Focus group interviews in nursing research: Part 1. British Journal of Nursing, 22(1), 16–19. 10.12968/bjon.2013.22.1.16 23299206

[hsc13839-bib-0011] Flynn, M. , Keywood, K. , & Fovargue, S. (2003). Warning: Health ‘choices’ can kill. The Journal of Adult Protection, 5(1), 30–34. 10.1108/14668203200300005

[hsc13839-bib-0012] Green, J. , & Thorogood, N. (2014). Qualitative methods for health research (3rd ed.). Sage.

[hsc13839-bib-0013] Guest, G. , Namey, E. , Taylor, J. , Eley, N. , & McKenna, K. (2017). Comparing focus groups and individual interviews: Findings from a randomized study. International Journal of Social Research Methodology, 20(6), 693–708. 10.1080/13645579.2017.1281601

[hsc13839-bib-0014] Hancock, D. (2010). Tame, messy and wicked risk leadership. Ashgate.

[hsc13839-bib-0015] Hart, E. , & Bond, M. (1995). Action research for health and social care. OUP.

[hsc13839-bib-0016] Hinsliff‐Smith, K. , Feakes, R. , Whitworth, G. , Seymour, J. , Moghaddam, N. , Dening, T. , & Cox, K. (2017). What do we know about the application of the mental capacity act (2005) in healthcare practice regarding decision‐making for frail and older people? A systematic literature review. Health & Social Care in the Community, 25(2), 295–308. 10.1111/hsc.12310 26611194

[hsc13839-bib-0017] House of Lords Select Committee on the Mental Capacity Act 2005 . (2014). Mental capacity act 2005: Post‐legislative scrutiny . HL139 HMSO. http://www.publications.parliament.uk/pa/ld201314/ldselect/ldmentalcap/139/139.pdf

[hsc13839-bib-0018] Jayes, M. , Austin, L. , & Brown, L. J. E. (2021). Supported decision‐making and mental capacity assessment in care homes: A qualitative study. Health & Social Care in the Community, 00, 1–9. 10.1111/hsc.13512 34250675

[hsc13839-bib-0019] Jayes, M. , Palmer, R. , Enderby, P. , & Sutton, A. (2019). How do health and social care professionals in England and Wales assess mental capacity? A literature review. Disability and Rehabilitation, 42(19), 2797–2808. 10.1080/09638288.2019.1572793 30739505

[hsc13839-bib-0020] Jenkins, C. , Webster, N. , Smythe, A. , & Cowdell, F. (2020). What is the nature of mental capacity act training and how do health and social care practitioners change their practice post‐training? A narrative review. Journal of Clinical Nursing, 29(13–14), 2093–2106. 10.1111/jocn.15256 32223040

[hsc13839-bib-0021] Jones, S. , Williams, B. , & Monteith, P. (2014). Decision making for refusals of treatment—A framework to consider. Journal of Paramedic Practice, 6(4), 180–186. 10.12968/jpar.2014.6.4.180

[hsc13839-bib-0022] Kamberelis, G. , & Dimitriadis, G. (2013). Focus groups: From structured interviews to collective conversations. Routledge.

[hsc13839-bib-0023] Kane, N. , Ruck Keene, A. , Owen, G. , & Kim, S. (2021). Applying decision‐making capacity criteria in practice: A content analysis of court judgments. PLoS ONE, 16(2), e0246521. 10.1371/journal.pone.0246521 33544766 PMC7864395

[hsc13839-bib-0024] Lauder, W. , Davidson, G. , Anderson, I. , & Barclay, A. (2005). Self‐neglect: The role of judgments and applied ethics. Nursing Standard, 19(18), 45–51. 10.7748/ns2005.01.19.18.45.c3785 15683055

[hsc13839-bib-0025] Madill, A. , & Gough, B. (2008). Qualitative research and its place in psychological science. Psychological Methods, 13(3), 254–271. 10.1037/a0013220 18778154

[hsc13839-bib-0026] Manthorpe, J. , Harris, J. , Burridge, S. , Fuller, J. , Martineau, S. , Ornelas, B. , Tinelli, M. , & Cornes, M. (2021). Social work practice with adults under the rising second wave of Covid‐19 in England: Frontline experiences and the use of professional judgement. The British Journal of Social Work., 51, 1879–1896. 10.1093/bjsw/bcab080

[hsc13839-bib-0027] Marshall, H. , & Sprung, S. (2016). Community nurse’s knowledge, confidence and experience of the mental capacity act in practice. British Journal of Community Nursing, 21(12), 615–622. 10.12968/bjcn.2016.21.12.615 27922781

[hsc13839-bib-0028] Martineau, S. , Cornes, M. , Manthorpe, J. , Ornelas, B. , & Fuller, J. (2019). Safeguarding, homelessness and rough sleeping: An analysis of safeguarding adults reviews. NIHR. 10.18742/pub01-006

[hsc13839-bib-0029] Mason, K. , & Evans, T. (2020). Social work, inter‐disciplinary cooperation and self‐neglect: Exploring logics of appropriateness. British Journal of Social Work, 50(3), 664–681. 10.1093/bjsw/bcz031

[hsc13839-bib-0030] Mental Health Foundation . (2010). Mental capacity assessments: Working in practice? Initial findings from the Mental Health Foundation’s assessment of mental capacity audit . https://www.mentalhealth.org.uk/publications/listing?search=capacity&field_focus_section_target_id=All&page=2

[hsc13839-bib-0031] NHS Digital . (2021). Safeguarding adults, England, 20–21, Experimental statistics. https://digital.nhs.uk/data‐and‐information/publications/statistical/safeguarding‐adults/2020‐21

[hsc13839-bib-0032] Preston‐Shoot, M. (2020). Safeguarding adult reviews: Informing and enriching policy and practice on self‐neglect. Journal of Adult Protection, 22(4), 199–215. 10.1108/jap-02-2020-0003

[hsc13839-bib-0033] Preston‐Shoot, M. , Braye, S. , Preston, O. , Allen, K. , & Spreadbury, K. (2020). Analysis of safeguarding adult reviews, April 2017–March 2019 . https://www.local.gov.uk/publications/analysis‐safeguarding‐adult‐reviews‐april‐2017‐march‐2019

[hsc13839-bib-0034] Pritchard‐Jones, L . (2020). The inherent jurisdiction of the high court: Practice guidance . https://www.researchinpractice.org.uk/media/4683/inherent_jurisdition_pg_web.pdf

[hsc13839-bib-0035] Ratcliff, D. , & Chapman, M. (2016). Health and social care practitioners’ experiences of assessing mental capacity in a community learning disability team. British Journal of Learning Disabilities, 44(4), 329–336. 10.1111/bld.12172

[hsc13839-bib-0036] Rogers, J. , & Bright, L. (2019). Assessments of mental capacity: Upholding the rights of the vulnerable or the misleading comfort of pseudo objectivity? The Journal of Adult Protection, 21(2), 74–84. 10.1108/jap-10-2018-0026

[hsc13839-bib-0037] Ruck Keene, A. (2021). Towards more satisfactory capacity determinations . https://www.researchinpractice.org.uk/adults/news‐views/2021/may/towardsmore‐satisfactory‐capacity‐determinations/

[hsc13839-bib-0038] Ruck Keene, A. , Butler‐Cole, V. , Allen, N. , Bicarregui, A. , Kohn, N. , & Akhtar, S. (2016). A brief guide to carrying out capacity assessments . http://www.39essex.com/wp‐content/uploads/2016/08/Capacity‐Assessments‐Guide‐August‐2016.pdf

[hsc13839-bib-0039] Ruck Keene, A. , Kane, N. B. , Kim, S. Y. H. , & Owen, G. S. (2019). Taking capacity seriously? Ten years of mental capacity disputes before England’s court of protection. International Journal of Law and Psychiatry, 62, 56–76. 10.1016/j.ijlp.2018.11.005 30616855 PMC6338675

[hsc13839-bib-0040] Scott, J. , Weatherhead, S. , Daker‐White, G. , Manthorpe, J. , & Mawson, M. (2020). Practitioners’ experiences of the mental capacity act: A systematic review. Journal of Adult Protection, 22(4), 35–54. 10.1108/jap-02-2020-0005

[hsc13839-bib-0041] Shepherd, V. , Griffith, R. , Sheehan, M. , Wood, F. , & Hood, K. (2018). Healthcare professionals’ understanding of the legislation governing research involving adults lacking mental capacity in England and Wales: A national survey. Journal of Medical Ethics, 44, 632–637. 10.1136/medethics-2017-104722 29695407 PMC6119350

[hsc13839-bib-0042] Social Care Institute for Excellence . (2017). Assessing capacity . https://www.scie.org.uk/mca/practice/assessing‐capacity

[hsc13839-bib-0043] Social Care Institute for Excellence .(2020). Mental Capacity Act 2005 at a glance . https://www.scie.org.uk/mca/introduction/mental‐capacity‐act‐2005‐at‐a‐glance

[hsc13839-bib-0044] Taylor, H. (2015). Improving the efficacy of the mental capacity act. Nursing Times, 111(27), 20–23.

[hsc13839-bib-0045] Willner, P. , Bridle, J. , Price, V. , Dymond, S. , & Lewis, G. (2013). What do NHS staff learn from training on the mental capacity act (2005)? Legal & Criminological Psychology, 18(1), 83–101. 10.1111/j.2044-8333.2011.02035.x

